# Case of Fibroid Tumour of the Upper Jaw

**Published:** 1870-02

**Authors:** 


### ARTICLE VIII.

### *Case of Fibroid Tumour of the Upper Jaw.*

Dr Ashhurst exhibited the specimen, and read the following history before the "Pathological Society,"

R. H., aged 17, a native of this country, and an iron-worker by trade, was admitted to the Episcopal Hospital on Jan. 20, 1869. It was impossible to obtain any satisfactory account of the previous history of this case, but we learned that he had noticed some obstruction of the right nostril for several months, and latterly the cheek had become swollen below and in front of the malar protuberance. On examination the hard palate was found depressed, and in one part much thinned, so that the presence of fluid above it was easily recognized. There was no enlargement in the region of the canine fossa, no encroachment upon the orbit, and scarcely any on the nostril. It was evident that there was dropsy of the antrum, and the swelling of the cheek—which the patient said was not constant—was supposed to be either due to inflammatory thickening, or possibly a new growth unconnected with the maxillary affection. A trocar and canula were introduced through the bulging fluctuating surface of the hard palate, and a considerable quantity, probably a fluid ounce, of serous fluid deeply tinged with blood, evacuated with immediate relief to the symptoms.

The amendment in the patient's condition was, however, of but short duration, and in the early part of February profuse hæmorrhage from the posterior nares occurred on three several occasions. He now mentioned (a fact of which we had before been unaware), that similar hæmorrhages had occurred before he sought admission to the hospital. Examination at this time revealed the existence of a solid tumour, the presence of which when smaller had been masked by the fluid accumulation in the antrum. It was now perceived that the entire alveolar border of the jaw was displaced downwards and outwards, and the finger introduced behind the soft palate recognized a tumor projecting backwards into the pharynx. The soft palate itself was manifestly depressed by the superincumbent mass. No connection could be traced between the maxillary tumour and the swelling in the cheek, which I thought I could isolate from the larger mass, getting my finger as I supposed behind and entirely around it.

As the patient was rapidly becoming anæmic from repeated hæmorrhages, and as the dyspnœa was constantly increasing, it was evident that operative interference was urgently demanded. Accordingly, on Feb. 18, with the approval and assistance of my colleagues, I removed the greater portion of the right upper maxilla (leaving only the orbital plate, which was perfectly normal), and then proceeded to enucleate the tumor with its several ramifications as rapidly as possible. The external incisions were through the median line of the lip along the ala of the nose, and transversely below the orbital ridge. The vessels of the flaps were tied as they were divided. The bone sections were made with strong cutting pliers, and the jaw as well as the deep-seated portions of the tumour wrenched out with the " lion-jawed " forceps of Sir Wm. Fergusson. The last portion enucleated brought with it a bulbous mushroom-like projection, which proved to be the tumour that had been felt in the cheek. The whole growth was very vascular, and a great deal of blood was lost during the latter stages of the operation. The lips of the wound were approximated and the patient rallied from the chloroform anæsthesia sufficiently to answer questions, but never fairly reacted, and died about two hours after the operation.

The specimen shows the greater part of the tumour and the portion of jaw removed. The antrum is greatly enlarged. The morbid growth appears to have originatep from the posterior wall of the antrum, pressing down the hard palate, and partially displacing the alveolar ridge; it seems then to have escaped into the middle meatus of the nose, and to have grown rapidly upwards (towards the sphenoid and ethmoid bones), backwards into the pharynx, and finally to have crept outwards through the pterygo-maxillary fissure, making its appearance below the malar bone, in the soft structures of the cheek. The tumour, under the microscope, showed a large number of small vessels, with fibres, cells, and free nuclei. Even in its present state, shrunken from preservation in alcohol, the tumour is the size of a hen's egg, and before removal, when distended with blood was very much larger—much larger, I may add, than any of us supposed before beginning the operation.

I may perhaps be pardoned for alluding to one or two points of clinical, rather than pathological, interest. The unfortunate result in this case, was I think, manifestly due to the combined effects of shock and hæmorrhage. The patient was under the influence of chloroform for a considerable time (and very thorough anæsthesia is requisite for operations on the jaw), but it is to be remembered that without anæsthesia, such an operation would be well nigh impossible. The immediate proximity of the tumour to the base of the skull is a very serious complication, and has not unfrequently been the cause of rapid sinking after the operation. This occurred in a case of Liston's. (*Erichsen's Surgery*, p. 863.) Mr. Hewet attempted to remove a fibrous tumour from behind the jaw by what has been called osteoplastic resection; the patient died before the operation could be terminated. (*Med.-Chir. Trans.*, vol. xxxiv. p. 43.) Quite recently, Sir Wm. Fergusson in an operation for tumour connected with the upper jaw, found the hæmorrhage so profuse that, to prevent a similar untoward result, he thought it better to close the wound, purposing to conclude the operation on another occasion. (*Med. Times and Gazette*, Aug. 22, 1868.) Careful and repeated consideration and criticism of the case I have just narrated, have failed to suggest any plan by which the operation might have been rendered more likely to terminate well. Sir Wm. Fergusson's plan would not have answered in this case, for the tumour itself was so vascular, that to leave any portion in position, would have exposed the patient to an almost certainty of continuous bleeding. Ligature of one or both common carotids would of course be out of the question. Possibly, had the operation been done earlier, the patient being less anæmic at the start, and the tumour smaller, a more favorable result may have been attained. The case was one that gave me a great deal of anxiety and care during the whole time it was under my observation, and, I think, should be placed on record, if for nothing else, as an illustration of the difficulties of diagnosis and the risks of treatment in all cases of tumour connected with the upper jaw and the base of the skull.—*Am. Jour. of the Med. Sciences.*

---

